# The Impact of Distal Influences and Proximal Resources on the Mental Health of African American Older Adults: Findings From the Georgia Centenarian Study

**DOI:** 10.1093/geroni/igaa046

**Published:** 2020-09-18

**Authors:** Meneka C Johnson Nicholson, Peter Martin, Megan Gilligan, Carolyn E Cutrona, Daniel W Russell, Tom J Schofield, Leonard W Poon

**Affiliations:** 1 Operations Department, Rural Health Medical Program, Inc., Selma, Alabama; 2 Department of Human Development and Family Studies, Iowa State University, Ames; 3 Department of Psychology, Iowa State University, Ames; 4 Research Division, Los Angeles County Probation Department, California; 5 Institute of Gerontology, University of Georgia, Athens

**Keywords:** Minority issues, Octogenarians, Oldest old, Psychosocial, Self-rated health, Sexagenarians, Social networks, Social support

## Abstract

**Background and Objectives:**

Over the years, a large amount of research has been devoted to the investigation of factors that led to mental health outcomes in older adults. For African American older adults, their lived experiences place them at high risk for mental health problems. The purpose of this study was to examine the impact of early life influences (i.e., education, childhood life events, and childhood financial well-being) and present psychosocial resources (i.e., individual, financial, and social) on current mental health outcomes in a sample of African American older adults in their 60s, 80s, and 100s.

**Research Design and Methods:**

Using data from the Georgia Centenarian Study, 125 participants were interviewed about their mental health, resources, and early life influences.

**Results:**

A structural equation model was tested and resulted in a good fit. Results indicated that the more social resources African American older adults had available, the lower the number of depressive symptoms they reported. African Americans with higher levels of financial well-being during childhood reported higher self-rated mental health. Older adults had higher levels of financial resources. Level of education showed a positive relationship with financial resources. Indirect effects of distal influences on health outcomes via current resources were not found.

**Discussion and Implications:**

The findings are of direct practical relevance and can be used to more readily identify older African Americans who may be susceptible to poorer mental health outcomes based upon the impact of their unique distal and proximal psychosocial resources.

**Translational Significance:** This study was undertaken to determine the impact of distal and proximal influences on mental health outcomes in late life. This analysis suggests that the self-perception of financial well-being during childhood and current social resources shapes mental health outcomes in late adulthood, particularly in African Americans older than the age of 60. These findings further emphasize the significance of the lasting effects of the overall lived experiences of African American older adults as these experiences place them at high risk for mental health problems. Therefore, through the use of measurement tools validated for diverse populations, it is necessary to understand how these cumulative experiences affect the resources African American older adults may possess and/or lack in later life, in order to best aid them in acquiring and in perceiving themselves to have had optimal aging experiences, as positive self-perspectives have shown to predict longevity and health.

Mental health is a fundamental component of well-being and plays an integral role in the process of successfully aging. Mental health outcomes have been widely studied within aging and have been associated with a myriad of factors. In recent years, researchers have become increasingly interested in examining the influence of early life factors and psychosocial resources on the various aspects of later life (Carr, 2019; Kok et al., 2017). So far, this method has typically been applied to the direct effects of early life factors and psychosocial resources, as far too little attention has been paid to the mediation pathways that connect early life experiences to the present state of resources and mental health outcomes in older adults. As the population continues to age and declines in physical health and resources remain as natural progressions of aging, it should be anticipated that mental health issues will be more prevalent because these factors affect mental health (Administration for Community Living, 2016). It would be advantageous to investigate the impact of distal and psychosocial resources on later life mental health, especially within those individuals who are able to obtain the status as the oldest old in order to best understand the means that are needed in which to best support them.

As the aging population becomes more diverse, it is important to explore the racial differences in mental health outcomes due to distal and proximal conditions and experiences. In the black American population, mental health issues are highly prevalent due to factors that stem from the lived black experience, such as the effects of discrimination and institutionalized racism, which can lead to unfavorable circumstances such as socioeconomic immobility, differential resource access, and negative self-evaluations (Williams & Williams-Morris, 2000). Naturally, an increase in age leads to an increase in the number of opportunities for negative experiences to have occurred across the life span. In addition, older African Americans are less likely to access mental health services across the life course (Kawaii-Bogue et al., 2017) and are therefore more vulnerable to mental health problems in old age due to cumulative stressors. Thus, it is of interest to know whether early experiences influence present levels of psychosocial resources and its effect on the outcomes of mental health specifically in older African Americans.

This study builds on Martin and Martin’s (2002) resource model of developmental adaptation and contributes a model for understanding the impact of distal and proximal psychosocial resources on mental health in the African American older adult population. The hypothesized model posits that mental health, as a developmental outcome variable, is influenced by past experiences (i.e., education, childhood life events, and childhood financial well-being) and present psychosocial resources (i.e., individual, financial, and social resources) among older African Americans. Until now, this model had not been tested exclusively within an ethnic minority sample.

## Mental Health in Older African Americans

There has been a longstanding investigation of factors that lead to mental health outcomes in older adulthood in African Americans (Taylor & Chatters, 2020). In many empirical studies, mental health is typically conceptualized and operationalized as a combination of psychological distress, mental illness, or psychological disorders. Depressive symptoms and respondent-assessed health status have been found to be key indicators of general well-being and mental health among older adults (Administration for Community Living, 2016) and are the primary foci in the current study.

### Risk Factors for African Americans Mental Health in Older Adulthood

Depression is a major public health concern and a common occurrence among African Americans and older adults as they are often underdiagnosed, lack access to, and/or forgo medical treatment (Kawaii-Bogue et al., 2017). Psychological distress often is linked to life stressors (e.g., illness, crime, and poverty). In older African Americans, lower education levels, functional disability, material hardships, stressful life events, the role of discrimination, social support, networks, and interactions have all been found to be predictors of depressive symptoms and psychological distress across the literature (Evans et al., 2019; Lincoln et al., 2010). Additionally, prior research revealed that certain characteristics such as being female, unmarried, older, and having a low socioeconomic status (SES) increase vulnerability to depressive symptoms and psychological distress (Lincoln et al., 2010).

### Measurement of Mental Health

Over the years, a large amount of research has been devoted to the measurement of mental health. Previous research has varied on assessment tools used to measure mental health outcomes. The work of Barg et al. (2006) acknowledged the fact that it is sometimes difficult to identify mental health issues in older adults as they may not describe their experiences as such. In their 2009 study, Pedraza et al. (2009) found this issue to be further compounded within the older African American population. Barnes and Bates (2017) conducted a literature review and found that in the majority of the studies, African Americans reported lower rates of major depression than Caucasians, yet had higher rates of symptoms of depression and distress. Due to findings such as these, numerous scholars have openly questioned whether survey instruments that assess mental health actually measure symptomatology accurately across various ethnic groups and equally across the life course (Alang, 2018; Barnes & Bates, 2017). Researchers have also considered the implications of the applicability of largely recognized survey instruments that have been validated among middle-aged whites, within diverse populations (Alang, 2018; Pedraza et al., 2009).

Past studies have yielded some important insights into the issue of the variations in which late-life mental health issues clinically present in African Americans, as well as other ethnic minority groups (Alang, 2018; Taylor & Chatters, 2020; Taylor et al., 2018). As a whole, African Americans tend to report more somatic symptoms and less dysphoria (Taylor & Chatters, 2020). Although there is broad agreement that African Americans experience depressive symptoms, it remains controversial whether older African Americans actually have more or fewer symptoms than whites as studies have produced mixed results as these differences persisted regardless if depression was measured by a cutoff score (Barnes & Bates, 2017; Lincoln et al., 2010). Barnes and Bates (2017) acknowledged that some of the incongruency could be due to possible differential item functioning between blacks and whites in diagnostic interviews.

An additional assessment method that has drawn considerable attention is self-reported mental health. Self-assessed mental health is a significant predictor of mortality in older adults and has also been found to measure not only current mental health status but is predictive of future mental health status (Jylhä, 2009). Numerous scholars recognized the importance of understanding the dynamics which influence the self-assessment of mental health outcomes (Benyamini et al., 2000; Jylhä, 2009). Research has found that although the typical negative indicators of health (e.g., disability, disease history, and medication) affect perceived mental health, there are psychosocial indicators, such as social resources or coping, which also show strong effects on an individual’s perception of mental health (Benyamini et al., 2000). Further research is needed to clarify the influence of psychosocial factors, from past and present, on mental health.

Although self-rated mental health is supported as a valid indicator, it is not all encompassing of mental health disorders. Hence, it is necessary to utilize additional measures to capture a broader picture of the mental health status of older adults. The current study adopts a psychosocial perspective in the investigation of distal and proximal influences on perceived mental health outcomes of depressive symptoms and mental health status in a sample of African American older adults. Distal influences included in this study are childhood life events, family financial well-being in childhood, and education. Social resources, financial resources, and coping behaviors are included as proximal resources.

## Distal Events

### Childhood Life Events

Research has produced inconsistent results on the effects of childhood life events and adversity on older adult mental health. It can be stated that one of the most traumatic events a child can experience is the death of a parent. The early loss of a parent can negatively affect the extent to which the child’s cognitive, emotional, social, and financial needs are met (Luecken & Roubinov, 2012). Research in the last decades found early loss of parents resulted in poorer mental health outcomes such as depression and psychiatric disorders in middle-aged and older adults as parental death in childhood had more impact throughout the life course than experiencing a parental death in adulthood (Kessler et al., 2010; Luecken & Roubinov, 2012). Kessler et al. (2010) found that childhood adversities, such as parental death and family economic adversity, were significantly associated with elevated risk for mental health disorders. In the current study, early traumatic losses were tested as predictors of mental health outcomes in later life.

### Childhood Financial Well-Being

Childhood financial well-being is vital as the conditions and experiences that occur during childhood establish the foundations of development that affect individuals across the life span (Luo & Waite, 2005). Economic hardships in early life can hinder access to higher levels of social and financial resources as well as affect physical and mental health in later life (Szanton et al., 2010). Luo and Waite (2005) studied the impact of childhood and adulthood SES on mental health outcomes and showed that lower childhood SES was associated with worse mental health in later life. More specifically, higher childhood SES predicted fewer depressive symptoms, with no clear mental health differences found between those from average and wealthy families. The current study sought to examine the impact of childhood SES, more specifically financial well-being, on mental health in late life.

### Education

Education is a highly influential variable on mental health outcomes because it is associated with the type and frequency of health behaviors an individual chooses to participate in (Adler et al., 1994). Higher education served as a protective factor against depression for older adults, whereas more incidents of depression were reported from those with less education (Fiske et al., 2009). The current study sought to concurrently examine three early life factors: early traumatic life events, childhood financial well-being, and education.

## Proximal Resources

### Social Resources

The relationship between social resources and mental health has been widely studied with consistent findings supporting its physiological and psychological benefits. Components of social resources have been studied as protective factors of the negative effects of stress on mental health. Social support and social networks can serve as protective factors in preventing depressive symptoms in African Americans (Taylor et al., 2015). An individual’s relationship within a social network has been found to be one of the most important predictors of depressive symptomology in older adults and African Americans (Fiori et al., 2006). Fiori et al. (2006) found that individuals who categorized their social networks as “diverse” made up primarily of “friends” or made up primarily of “nonfriends” reported significantly lower depressive symptoms than those from networks categorized as restricted. Perceived support also mediated the relationship between network type and depressive symptoms (Fiori et al., 2006). It has been suggested that, for older adults, perception of their networks is important for their mental health as networks that are not family only or restricted provide an added benefit of protection. African Americans report using informal social support networks as one of their most common methods of coping in stressful situations (Nguyen et al., 2016; Taylor et al., 2015). The quantity, quality, and type of social support affect mental health outcomes (Benca-Bachman et al., 2020; Nguyen et al., 2016). The current study examines social resources as predictors of mental health outcomes in later life.

### Financial Resources

Empirical evidence appears to confirm the notion that economic status, whether studied as wealth and income or financial hardship and strain, is associated with mental health across all stages of the life course. Luo and Waite (2005) found evidence that higher levels of income and wealth and lower levels of strain and hardship resulted in lower levels of depression. Previous studies have acknowledged the direct effects of financial strain on mental health; however, in a study of older African Americans, Lincoln (2007) pointed out that the effects of financial strain on mental health may not always be direct and may be mediated through other factors such as social interactions and coping mechanisms as a form of mastery. In the current study, past and present levels of SES are tested as predictors of mental health in later life.

### Active Coping

Coping is a very important behavior as it is seen as a process in which individuals choose to handle the stresses of life as a means to protect themselves. Previous studies have found a reciprocal relationship between coping and mental health as poor coping behaviors resulted in poorer mental health and poorer mental health resulted in poor coping behaviors (Holahan et al., 2005). Di Benedetto et al. (2014) studied the predictive effects of coping on mental health and showed that fewer coping resources predicted worse mental health and higher levels of depression. Coping also showed a significant indirect effect through depression on mental health (Di Benedetto et al., 2014). In older adults, loss of psychosocial resources such as coping predicted mental distress more than their medical conditions, demonstrating the importance of knowing how to manage stressors for good mental health (Ormel et al., 1997). The current study tested active coping as a predictor of mental health outcomes in later life.

## The Present Study

A major goal of this research was to present a strengths-based perspective on mental health among aging African Americans by highlighting the type and level of resources African American older adults possess. A key question that the current study was designed to address is the impact of psychosocial and financial resources on mental health outcomes in the lives of older African Americans. This is in contrast to much of aging research that considers racial differences in which ethnic minority older adults are compared to whites across a variety of variables, with whites considered as the normative group against which to evaluate study findings. A limitation of such research is that it does not take into account the unique characteristics that exist within the African American population (intragroup heterogeneity). It is the intent of this research to move away from framing minority aging as a deficient process and more toward understanding the complete aging experience of older African Americans. The objective of the current study was to assess the impact of distal and proximal influences on developmental mental health outcomes in later life.

We hypothesized that early life experiences and resources would affect mental health outcomes through the mediation of current resources. It is assumed that individuals’ developmental history affects their individual, social, and financial resources. In turn, perceptions of current psychosocial resources in one’s present life are expected to predict mental health outcomes for African American older adults. In our adapted framework of Martin and Martin’s model, early life influences such as life events, childhood financial well-being, and education, as well as the present level of coping, social and financial resources were investigated for their influence on depressive symptoms and perceived mental health in late adulthood. The model tested in this article viewed mental health as a function of individual, social, and financial resources, resources hypothesized as influenced by factors that occurred in early life.

## Method

Data for the current study come from the Georgia Centenarian Study (GCS). The GCS is an investigation of longevity and survival of the oldest old that took place from 1988 to 2009 in three study phases. Participants were drawn from a population-based sample of 701 community-dwelling and institutionalized centenarians (i.e., those aged 98 years and older), octogenarians (i.e., those aged 80–89 years), and sexagenarians (i.e., those aged 60–69 years) from the GCS. The sample consisted of two cohorts collected from two separate phases. Individuals born between 1881 and 1895 were part of Phase 1 and classified as the earlier cohort. The later cohort included persons born between 1901 and 1907 and was part of Phase 3. Phase 2 consisted of a longitudinal follow-up which was not considered in this study.

The analytic sample used in our investigation consists solely of African American older adults. Participants who were identified as cognitively unstable, as defined by a cutoff score of less than 17 on the Mini-Mental State Examination (MMSE) scale, were removed from analyses. In Phase 1, there were 321 total participants with 89 reported as African Americans. It should be noted that participants from Phase 1 were cognitively intact as measured by the MMSE and by ratings on the Global Deterioration Scale. In Phase 3, there were 380 total participants with 81 reported as African Americans. Of the 81 participants, 36 had an MMSE score of 17 or greater. The 89 Phase 1 participants were combined with the 36 participants from Phase 3 for a combined sample of 125 participants. Four percent of our remaining sample (five participants) lived within a care facility whereas the others were community dwelling. The overall sample is somewhat small relative to the variables used in our analyses, but the sample is unique as it includes African Americans in old and very old age.

### Measures

#### Distal variables

Education was measured in intervals, ranging from 0 years of schooling to the postgraduate college level. Childhood financial well-being was assessed by asking participants to report their perceptions of their early life SES. Participants were asked how they rated their childhood financial well-being (1 = *pretty well off financially*, 2 = *about average*, 3 = *poor*, and 4 = *it varied*). Life events consisted of a yes or no list of three early death experiences, including the death of the participant’s father, mother, and friend. A death that occurred prior to age 18 was classified as an early life experience.

#### Individual resources

Individual resources were assessed with one measure of active coping (α = 0.52). Active coping items included whether or not participants would say they “try hard to make things work,” “talk with their family about problems,” and “talk to their doctor about problems.” A mean score across the three items was computed.

#### Social resources

Social resources were assessed with three items from the Older Americans Resources and Services (OARS) Multidimensional Functional Assessment Questionnaire (Fillenbaum, 1988). Participants were assessed regarding the extent and type of contact they had with others. Participants were asked, “What is the number of people you know well enough” (0 = *none*, 1 = *one or two*, 2 = *three or 4*, and 3 = *five or more*) and to indicate “How many times you talked to someone in the past week” (0 = *none*, 1 = *once per week*, 2 = *2–6 times per week*, and 3 = *once a day or more*) and “How many times you spent time with someone in the past week” (0 = *none*, 1 = *once per week*, 2 = *2–6 times per week*, and 3 = *once a day or more*). We created a count variable and used the mean of the three items as the social resource variable. This measure is best understood as an assessment of the type of structure of the social resources older adults have available to them.

#### Financial resources

Perceived economic status (α = 0.81) was assessed using the economic scales from the Duke OARS (Fillenbaum, 1988). Participants self-assessed their financial resources using the following *yes or no* questions: “Are your assets and financial resources sufficient to meet emergencies?” and “At the present time, do you feel that you will have enough for your needs in the future?” Participants were also questioned as to “whether their expenses were so heavy that they”: *cannot meet the payments*, *can barely meet the payments*, or *payments are no problem*. Lastly, respondents answered to “How well does the amount of money you have take care of your needs?”: *very well*, *fairly well*, or *poorly*. Items were combined to construct a mean scale of perceived economic status.

#### Mental health

Mental health was assessed using measures of perceived mental health and depressive symptoms. Perceived mental health was assessed by one item from the OARS Multidimensional Functional Assessment Questionnaire that asked participants, “How would you rate your mental and emotional health at this present time?” (0 = *poor*, 1 = *fair*, 2 = *good*, and 3= *excellent*). Depressive symptoms were assessed by the Geriatric Depression Scale that included 15 dichotomous (yes or no) items (α = 0.77). Participants were asked if they had experienced any of the depressive symptoms during the past week, with higher scores indicating higher depressive symptoms (0–15). Questions included, “Do you feel that your life is empty,” “Do you feel lonely or remote,” and “Do you feel pretty worthless the way you are now?”

#### Control variables

All analyses controlled for gender (0 = *male*, 1 = *female*), age category (*sexagenarians* = 1, *octogenarians* = 2, *centenarians* = 3), and cognitive functioning as assessed by the MMSE, with scores ranging from 17 to 30.

### Statistical Analyses

The analyses proceeded in three specific steps. First, we computed descriptive analyses (i.e., means and standard deviations) for the study variables using SPSS. Second, we computed bivariate correlations for the study variables, also in SPSS. Third, the hypothesized model was evaluated using Mplus Version 7 (Muthén & Muthén, 1998–2012). Using a structural equation modeling approach is appropriate for the current study because it allows for an overall evaluation of the hypothesized model, compares the model to plausible alternatives, and reports how well the hypothesized model fits with the collected data, as well as examines the interrelationships of relevant variables. The fit of the hypothesized model was evaluated with standard fit indices (i.e., χ ^2^, comparative fit index [CFI], the root mean square error of approximation [RMSEA], and the standardized root mean square residual [SRMR]). A second (modified) model was computed, allowing for direct effects from exogenous to outcome variables. Finally, a parsimonious model was tested in which nonsignificant paths were deleted to assess whether the parsimonious model would fit as well as the modified hypothesized model. These models were compared with a χ ^2^ difference test.

## Results

The majority of the sample were in the octogenarian age group (*M* = 2.26, *SD* = 0.82, range = 1–3), female (*M* = 0.66, *SD* = 0.48, range = 0–1), and bordering normal cognition levels (*M* = 24.63, *SD* = 3.40, range = 17–30). Older African Americans had lower levels of education (*M* = 3.59, *SD* = 2.33, range = 1–8), childhood economic well-being (*M* = 2.06, *SD* = 1.07, range = 1–4), and early life events (*M* = 0.43, *SD* = 0.60, range = 0–2). They were fairly high in financial and social resources (*M* = 1.15, *SD* = 0.42, range = 0.25–2; *M* = 2.36, *SD* = 0.51, range = 0–3), but lower in active coping behaviors (*M* = 0.82, *SD* = 0.28, range = 0–1). They perceived themselves to be in good mental health and low in depressive symptoms (*M* = 1.88, *SD* = 0.67, range = 0–3; *M* = 0.19, *SD* = 0.17, range = 0–1). Structural equation modeling was used to estimate the effects of age, gender, level of cognitive coping, social resources, and financial resources. In addition, the effects of these measures on depressive symptoms and the perceived mental health rating were estimated. The full structural model was tested using Mplus.

Correlations were found among the study variables. Cognition and educational level both were associated with all three resources: social resources (*r* = 0.21, *p* < .05; *r* = 0.20, *p* < .05), financial resources (*r* = 0.25, *p* < .01; *r* = 0.41, *p* < .001), and active coping (*r* = 0.25, *p* < .01; *r* = 0.21, *p* < .05). Depressive symptoms correlated with level of cognition, *r* = −0.22, *p* < .05, and education, *r* = −0.24, *p* < .01. Financial resources negatively correlated with depressive symptoms, *r* = −0.19, *p* < .05. Financial condition during childhood significantly (*p* < .001) correlated with perceived mental health (*r* = 0.31); however, it was not significantly correlated with depressive symptoms (*r* = −0.10, *p* = .29).

The overall fit of the hypothesized model was χ ^2^ (7) = 14.88, *p* = .04, suggesting that the fit to the data was not optimal. Alternative fit indices also suggested an inadequate fit, CFI = 0.89, SRMR = 0.04, and RMSEA = 0.10. Path coefficients are displayed in Table 1. Modification indices were inspected to improve the model. Modification indices suggested a direct path from childhood financial condition to perceived mental health. This modification resulted in a model fit of χ ^2^ (6) = 2.70, *p* = .85, suggesting a reasonable fit to the data. Alternative fit indices also suggested an acceptable fit, CFI = 1.0, SRMR = 0.02, RMSEA = 0.00. The results of the structural model are summarized in Table 2.

**Table 1.** Summary of Multiple Regression Analyses Hypothesized Model for Variables Predicting Mental Health (*N* = 106)

	*B*	*SE*	β
Social resources			
Age	−0.09	0.07	−0.14
Gender (female)	0.14	0.11	0.13
Cognition	0.02	0.02	0.11
Education	0.04	0.03	0.15
Childhood financial well-being	−0.04	0.05	−0.08
Childhood life events	−0.04	0.08	−0.05
Financial resources			
Age	0.13	0.06	0.24*
Gender (female)	0.03	0.08	0.03
Cognition	0.00	0.02	0.03
Education	0.09	0.02	0.48***
Childhood financial well-being	0.05	0.04	0.11
Childhood life events	−0.05	0.06	−0.07
Active coping			
Age	−0.01	0.04	−0.04
Gender (female)	0.09	0.06	0.14
Cognition	0.01	0.01	0.16
Education	0.03	0.02	0.29*
Childhood financial well-being	0.01	0.03	0.03
Childhood life events	−0.06	0.04	−0.12
Social resources	0.00	0.05	0.00
Financial resources	−0.22	0.07	−0.34**
Depressive symptoms			
Age	0.02	0.02	0.09
Gender (female)	0.01	0.04	0.06
Cognition	−0.01	0.01	−0.13
Active coping	0.05	0.06	0.08
Social resources	−0.08	0.03	−0.23*
Financial resources	−0.06	0.04	−0.16
Perceived mental health			
Age	−0.06	0.10	−0.07
Gender (female)	−0.01	0.15	−0.00
Cognition	−0.03	0.02	−0.16
Active coping	0.01	0.25	0.01
Social resources	0.15	0.14	0.12
Financial resources	0.18	0.16	0.11
*χ* ^2^	14.88	7 *df*	0.04
RMSEA	0.10		
CFI	0.89		
SRMR	0.04		

*Note:* CFI = comparative fit index; RMSEA = root mean square error of approximation; SRMR = standardized root mean square residual.

*p < .05, **p < .01, ***p < .001.

**Table 2.** Summary of Multiple Regression Analyses of Hypothesized Model With Modifications for Variables Predicting Mental Health (*N* = 108)

	*B*	*SE*	β
Social resources			
Age	−0.09	0.07	−0.14
Gender (female)	0.14	0.11	0.12
Cognition	0.02	0.02	0.11
Education	0.04	0.03	0.16
Childhood financial well-being	−0.04	0.05	−0.09
Childhood life events	−0.04	0.08	−0.05
Financial resources			
Age	0.13	0.06	0.24*
Gender (female)	0.03	0.08	0.03
Cognition	0.01	0.02	0.04
Education	0.09	0.02	0.48***
Childhood financial well-being	0.04	0.04	0.11
Childhood life events	−0.05	0.06	−0.07
Active coping			
Age	−0.01	0.04	−0.03
Gender (female)	0.09	0.06	0.14
Cognition	0.01	0.01	0.16
Education	0.03	0.02	0.29*
Childhood financial well-being	0.01	0.03	0.03
Childhood life events	−0.06	0.04	−0.12
Social resources	0.00	0.05	0.00
Financial resources	−0.22	0.07	−0.34**
Depressive symptoms			
Age	0.02	0.02	0.09
Gender (female)	0.02	0.04	0.06
Cognition	−0.01	0.01	−0.13
Active coping	0.05	0.06	0.08
Social resources	−0.08	0.03	−0.23*
Financial resources	−0.07	0.04	−0.16
Perceived mental health			
Age	−0.06	0.09	−0.08
Gender (female)	−0.06	0.14	−0.05
Cognition	−0.03	0.02	−0.15
Childhood financial well-being	0.20	0.06	0.32***
Active coping	−0.06	0.24	−0.03
Social resources	0.19	0.13	0.14
Financial resources	0.04	0.15	0.02
*χ* ^2^	2.70	6 *df*	*p =* .85
RMSEA	0.00		
CFI	1.00		
SRMR	0.02		

*Note:* CFI = comparative fit index; RMSEA = root mean square error of approximation; SRMR = standardized root mean square residual.

*p < .05, **p < .01, ***p < .001.

 Five direct effects were present. Higher levels of education predicted increased reports of active coping. A negative relationship was found between perceived economic status and active coping. The higher individuals perceived their economic status, the less active coping was used. Individuals in older age groups showed higher levels of perceived financial resources. Level of education was positively related to perceived financial resources. With regard to depressive symptoms, the more social resources older adults perceived they had available to them the lower the number of depressive symptoms they reported. One path was found to be significant from developmental history to perceived mental health. Adults with higher levels of financial well-being during childhood reported higher self-rated mental health. The indirect effects of distal influences on developmental health outcomes were examined using the bootstrap procedure but no indirect effects were found.

After a review of the overall conceptual model, several factors did not contribute significantly to the model as indicated by their standardized path coefficients. The following direct paths were found to be nonsignificant: from childhood financial condition, early life events, and social resources to active coping; from education, childhood financial condition, and early life events to social resources; from active coping and financial resources to depressive symptoms; and from active coping, social resources, and financial resources to perceived mental health. An alternative trimmed model that restricted these predictors to zero yielded the following results: χ ^2^ (*df* = 19) = 15.45, *p* = .69, CFI = 1.0, SRMR = 0.04, and RMSEA = 0.00. The path coefficients for this model are given in Table 3.

**Table 3.** Summary of Multiple Regression Analyses for Hypothesized Model for Variables Predicting Mental Health Outcomes (*N* = 108)

	*B*	*SE*	β
Social resources			
Age	−0.10	0.07	−0.17
Gender (female)	0.13	0.11	0.14
Cognition	0.03	0.02	0.15
Education	0.00	0.00	0.00
Childhood financial well-being	0.00	0.00	0.00
Childhood life events	0.00	0.00	0.00
Financial resources			
Age	0.14	0.06	0.27*
Gender (female)	0.04	0.08	0.03
Cognition	0.01	0.02	0.04
Education	0.09	0.02	0.50***
Childhood financial well-being	0.00	0.00	0.00
Childhood life events	0.00	0.00	0.00
Active coping			
Age	−0.01	0.04	−0.04
Gender (female)	0.09	0.06	0.16
Cognition	0.01	0.01	0.17
Education	0.03	0.01	0.28*
Childhood financial well-being	0.00	0.00	0.00
Childhood life events	0.00	0.00	0.00
Social resources	0.00	0.00	0.00
Financial resources	−0.20	0.07	−0.31**
Depressive symptoms			
Age	0.01	0.02	0.06
Gender (female)	0.02	0.04	0.05
Cognition	−0.01	0.01	−0.14
Active coping	0.00	0.00	0.00
Social resources	−0.06	0.03	−0.21*
Financial resources	0.00	0.00	0.00
Perceived mental health			
Age	−0.08	0.09	−0.10
Gender (female)	−0.03	0.14	−0.02
Cognition	−0.03	0.02	−0.13
Childhood financial well-being	0.19	0.06	−0.30***
Active coping	0.00	0.00	0.00
Social resources	0.00	0.00	0.00
Financial resources	0.00	0.00	0.00
*χ* ^2^	15.45	19 *df*	*p =* .69
RMSEA	0.00		
CFI	1.00		
SRMR	0.04		

*Note:* CFI = comparative fit index; RMSEA = root mean square error of approximation; SRMR = standardized root mean square residual.

*p < .05, **p < .01, ***p < .001.

**Figure 1.**
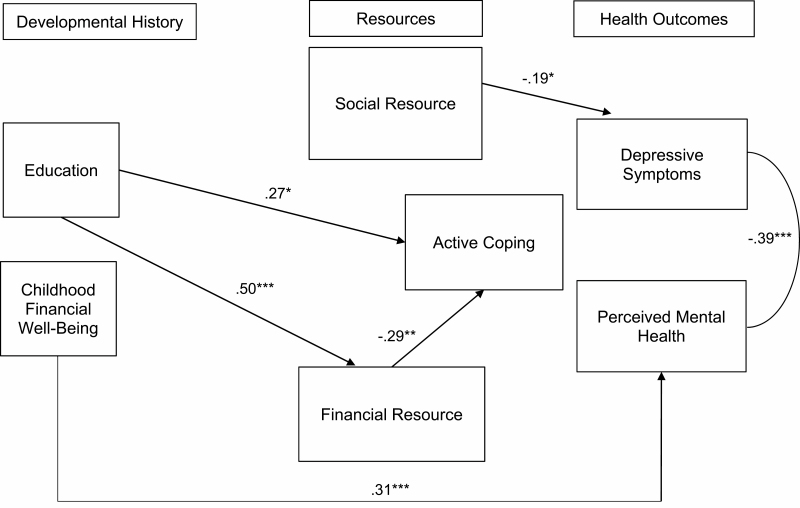
Parsimonious model. Controls: age group, gender, and cognition. Model fit, *χ*^2^ (*df* = 12) = 11.21, *p* = .51, CFI = 1.0; SRMR = 0.04; RMSEA = 0.00. Significant covariates: Age was predictive of financial resources (β = 0.26, *p <* .01). CFI = comparative fit index; RMSEA = root mean square error of approximation; SRMR = standardized root mean square residual. *p < .05, **p < .01, ***p < .001.

The parsimonious model was analyzed after the removal of the nonsignificant paths from the model. The overall fit of the resulting model was χ ^2^ (*df* = 12) = 11.21, *p* = .51, CFI = 1.0, SRMR = 0.04, and RMSEA = 0.00. This indicated that the reduced (“parsimonious”) model explains the data well. The model is depicted in Figure 1. Table 4 displays the fit indices of the first three tested models.

**Table 4.** Fit Indices of Tested Models

Model	*χ* ^2^	*df*	*p*	RMSEA	CI	CFI	Δχ ^2^	Δ*df*
1. Hypothesized model	14.88	7	0.04	0.10	0.02–0.18	0.89	—	—
2. Hypothesized model with added modification	2.70	6	0.85	0.00	0.00–0.07	1.00	12.18*	1
3. Restricted hypothesized model	15.45	19	0.69	0.00	0.00–0.07	1.00	12.75	13

*Note:* CI = confidence interval of the RMSEA value; CFI = comparative fit index; RMSEA = root mean square error of approximation; Δχ ^2^ = change in chi-square from the immediately preceding model; Δ*df* = change in degrees of freedom from the immediately preceding model.

*Denotes significant difference between model.

## Discussion

The aim of this study was to investigate the influence of early life factors and present psychosocial resources on depressive symptoms and perceived mental health in African American older adults. The hypothesized model did not show a good fit to the data. A direct path from childhood financial well-being resulted in a better fit to the model. Our findings show that high levels of social resources are significant predictors of low levels of depressive symptoms in older African Americans, which was similar to previous findings of social support as a protective factor against depression in African Americans and older adults (Taylor et al., 2015). Our data are consistent with results obtained by Benca-Bachman et al. (2020) and Nguyen et al. (2016) in which social support was associated with lower depression in African Americans. The available evidence seems to point to the conclusion that older adults who activate their social resources tend to experience lower levels of depressive symptoms. Our hypothesis that high levels of social resources predict high levels of mental health outcomes is partially supported. Although a negative association between social resources and depressive symptoms was observed, no relationship was found between social resources and perceived mental health. The lack of a significant association between social resources and perceived mental health may be due to lack of items assessing the quality of social resources, as the literature supports the existence of a stronger positive relationship between quality of support and mental health outcomes (Benca-Bachman et al., 2020; Nguyen et al., 2016).

Our findings show a positive association between financial well-being during childhood and mental well-being in later life. This finding is consistent with previous studies that found lower childhood SES associated with worse health outcomes in later life (Luo & Waite, 2005; Szanton et al., 2010). We expected that early childhood economic strain would affect mental health through the mediation of current financial strain as the literature has consistently supported the notion that adversity during childhood contributes to cumulative disadvantages across the life span, which is especially true of African Americans (Shuey & Willson, 2008). However, we did not find that the association between childhood financial well-being was due to indirect effects, through the mediation of current SES. A possible explanation for this finding could simply be that the long-term effects of lower levels of financial well-being during childhood on mental health may occur through biological, psychological, and/or social mechanisms that were not captured in the current study.

Educational attainment level was positively associated with perceived financial resources, which is in line with the general financial literature. Education showed a positive relationship to active coping resources, which is similar to findings obtained by Martin et al. (2001), who found a connection between education and coping behavior in older adults. Contrary to our expectations, analyses revealed that perceived financial resources had an inverse association with active coping. A possible interpretation may be that those who view themselves as having adequate financial resources may not experience certain stressful events due to their actual financial status that requires the utilization of active coping skills. This interpretation is consistent with previous findings which indicated that low financial strain resulted in the decreased need of support and higher feelings of mastery (Lincoln, 2007). It also suggests that financial resources may not be a good indicator for the use of coping skills, which is similar to previous literature that determined education to be a more reliable resource to determine the necessity of activating coping skills (Lincoln, 2007). The current study’s active coping variable consists of elements of support and mastery. Further consideration should take into account that even when older African Americans are found to objectively have low economic resources, financial strain may not always manifest as a stressor due to individual expectations (Lincoln, 2007). Thus, the current participants’ perceived economic status may be congruent with their financial expectations resulting in low or no stress further decreasing the need to activate specific coping skills.

In the current study, age was positively associated with perceived economic status. Research on age and economic status has produced mixed results. Similar to our findings, some studies have found that financial resources are perceived more positively with advanced age (Hansen et al., 2008). Prior studies found that older adults, although low in financial resources in terms of objective measures, rated themselves higher on their perceived economic conditions (Hansen et al., 2008). A related idea that might explain higher perceived economic status is that benefit programs, such as Social Security, provide higher income replacement and may give the perception of added financial resources. Many recipients are retirees with lower income, many of whom are minorities and women, which was consistent with our study sample.

The current study draws from the work of Martin and Martin (2002) who specified a model of developmental adaptation, which posits that past and current events and resources influence the health and well-being of individuals across the life span. This model, along with findings from our study, demonstrates the relevance of biographical variables to present resources to mental health status.

## Limitations and Conclusion

The present study had several limitations. We used cross-sectional data and therefore our findings cannot prove causation. The sample was somewhat small relative to the number of variables used in our analyses. However, our sample is unique as African Americans who survive into very old age (i.e., to become centenarians) are still rare, thus, our sample includes the most resilient African Americans who have been able to live well into older adulthood. Given the majority of study participants lived within the community, our findings may not be generalizable to older individuals who live in institutionalized settings. Future studies should include a larger number of participants who reside in skilled nursing facilities to differentiate between the varying mediators of mental health that may exist within community-dwelling versus institutionalized older African Americans to determine whether there are differences in the aging process. Additionally, study participants were all from a specific southeastern area of the United States. Due to selective mortality, this model may not fit more disadvantaged African Americans, African Americans who died at younger ages, or African Americans in other regions of the country. black Americans are not a monolithic group; hence, generalizations to the overall black population cannot be assumed beyond the subset examined. Future research will have to meet the challenge of recruitment of a more robust and diverse study sample in order to capture the variations that exist within the black American community as a whole.

The measures of mental health were self-assessments and may produce varied results if used with other samples as differences may exist for self-rated health outcomes due to cultural, age, or regional differences (Jylhä, 2009). The measurement of mental health outcomes in the current study also affects the conclusions that can be drawn. Barnes and Bates (2017) reminded us that differences in item functioning may exist within diagnostic interviews, whereas other scholars highlight the variations in how mental health issues present within this subgroup (Alang, 2018; Taylor et al., 2018). Although there is strong convergent evidence for the use of a single-item indicator of self-rated mental health, our measure of perceived mental health was not highly reliable. This decreases correlations with other variables. Furthermore, the measures of coping had relatively low reliability, which may have limited the magnitude of their associations with other variables. The measure of social resources assessed only the frequency of contact and network size, not the quality or supportiveness of relationships. Measures of perceived social resources correlated more strongly with mental health measures than measures of quantitative aspects of the social network. Future research will have to further our understanding of the validity of measurement tools within diverse ethnic populations.

Future studies will have to assess the extent to which the various types of distal and proximal psychosocial variables, in addition to biological variables, may serve as predictors of mental health outcomes in older African Americans. Although difficult to obtain due to the high mortality of individuals in late life, the collection of longitudinal data would be beneficial to determine possible causation of these distal and proximal influences. Future research also should take into account which developmental period is the most influential in later life. This begs the question of which life period(s), childhood, adolescence, young adulthood, or adult midlife, matter more in later life. Identifying this can lead to health and welfare policies that could focus on specific developmental stages.

In conclusion, understanding the impact of distal and proximal influences on mental health outcomes in late life for older African Americans is critical. An overall key finding is that an individual’s perception of their experiences and statuses, both past and present, have direct and indirect implications on their mental health and wellness. Given that older African Americans are less likely to be accurately diagnosed for mental health issues and referred to services, it is necessary for those who provide care to them to understand the significance of an individual’s self-perception of their early-and present life conditions and resources on their mental health outcomes in later life. Recognizing this importance will facilitate carers to aid aging African Americans in possessing, as well as, in perceiving themselves to have had optimal aging experiences, as positive perspectives have shown to predict longevity and health.
